# Fetal liver mesenchymal stem cells restore ovarian function in premature ovarian insufficiency by targeting MT1

**DOI:** 10.1186/s13287-019-1490-8

**Published:** 2019-11-29

**Authors:** Boxian Huang, Chunfeng Qian, Chenyue Ding, Qingxia Meng, Qinyan Zou, Hong Li

**Affiliations:** 1 Center of Reproduction and Genetics, Affiliated Suzhou Hospital of Nanjing Medical University, Suzhou Municipal Hospital, Suzhou, 215002 China; 2 State Key Laboratory of Reproductive Medicine, Nanjing Medical University, Nanjing, 210029 China

**Keywords:** Fetal mesenchymal stem cells, Premature ovarian insufficiency, Reactive oxygen species, MT1

## Abstract

**Background:**

With the development of regenerative medicine and tissue engineering technology, almost all stem cell therapy is efficacious for the treatment of premature ovarian failure (POF) or premature ovarian insufficiency (POI) animal models, whereas little stem cell therapy has been practiced in clinical settings. The underlying molecular mechanism and safety of stem cell treatment in POI are not fully understood. In this study, we explored whether fetal mesenchymal stem cells (fMSCs) from the liver restore ovarian function and whether melatonin membrane receptor 1 (MT1) acts as a regulator for treating POI disease.

**Methods:**

We designed an in vivo model (chemotherapy-induced ovary damage) and an in vitro model (human ovarian granulosa cells (hGCs)) to understand the efficacy and molecular cues of fMSC treatment of POI. Follicle development was observed by H&E staining. The concentration of sex hormones in serum (E2, AMH, and FSH) and the concentration of oxidative and antioxidative metabolites and the enzymes MDA, SOD, CAT, LDH, GR, and GPx were measured by ELISA. Flow cytometry (FACS) was employed to detect the percentages of ROS and proliferation rates. mRNA and protein expression of antiapoptotic genes (SURVIVIN and BCL2), apoptotic genes (CASPASE-3 and CASPASE-9), and MT1 and its downstream genes (JNK1, PCNA, AMPK) were tested by qPCR and western blotting. MT1 siRNA and related antagonists were used to assess the mechanism.

**Results:**

fMSC treatment prevented cyclophosphamide (CTX)-induced follicle loss and recovered sex hormone levels. Additionally, fMSCs significantly decreased oxidative damage, increased oxidative protection, improved antiapoptotic effects, and inhibited apoptotic genes in vivo and in vitro. Furthermore, fMSCs also upregulated MT1, JNK1, PCNA, and AMPK at the mRNA and protein levels. With MT1 knockdown or antagonist treatment in normal hGCs, the protein expression of JNK1, PCNA, and AMPK and the percentage of proliferation were impaired.

**Conclusions:**

fMSCs might play a crucial role in mediating follicular development in the POI mouse model and stimulating the activity of POI hGCs by targeting MT1.

## Background

Primary ovarian insufficiency (POI) or premature ovarian failure (POF) is a heterogeneous gynecological endocrine disorder. Cessation of ovarian function, followed by abnormal levels of gonadotropins and estrogen, are the characteristics of POI, which affects 4% of women under the age of 35 years old [1, 2]. The cause of POI is not fully understood; however, the decreased quality and quantity of follicles and oocytes in POI may be affected by abnormal genetic factors, iatrogenic factors (chemotherapy and radiotherapy process), metabolic factors, mitochondrial dysfunction, autoimmunity and environmental factors [3, 4]. Due to the mysterious and complicated nature of POI, there are a variety of approaches to treating POI, including hormonal replacement therapy, melatonin supplementation, dehydroepiandrosterone (DHEA) supplementation, immunomodulatory therapy, and stem cell therapy [5].

Given that hormone replacement therapy can only relieve menopausal symptoms, stem cell therapy provides a promising therapeutic approach to preserve fertility for POI patients who want to have a healthy baby. MSCs derived from bone (BMSCs) [6], adipose-derived stem cells (ADSCs) [7], human menstrual-derived stem cells [8], and perinatal stem cells (amniotic fluid, amniotic membrane) [9, 10], transplanted into a POI/POF animal model via intraperitoneal injection, intravenous injection, or microinjection needles at laparotomy to rescue ovarian function. In addition, conditioned medium of human amniotic epithelial cells (hAECs) [11] and exosomes from ADSCs [12] cocultured with human POI ovarian granule cells recovered ovarian function by promoting the proliferation rate and inhibiting the apoptosis rate. Moreover, some researchers showed that estrogen-sensitive epithelial cells [13] and granulose-like cells derived from human induced pluripotent stem cells (iPSCs) could rescue ovarian function [14].

Using healthy and functional cells to replace aged or damaged cells is the aim of stem cell therapy, and stem cells need to maintain cell viability, properties, and function before and after implantation in vivo. Some studies have indicated that fetal MSCs have longer lifespans than adult MSCs when they express similar immunomodulatory properties [15–17]. fMSCs from first trimester livers express many pluripotency stem cell markers and have greater telomerase activity than that of adult MSCs [18]. Recently, research has indicated that fetal liver (FL) MSC-derived exosomes might be a powerful immunomodulator to inhibit proliferation, activation, and cytotoxicity in NK cells through exosome-associated TGF [19]. Moreover, fetal MSCs have been detected in the bone marrow, blood, and liver in early gestation [18]. Compared to the fetal blood and bone marrow, fMSCs of the liver are easily obtained as a promising candidate. However, whether fMSCs of the liver improve ovarian fertility preservation and the potential molecular mechanisms are not clear.

Although almost all of the studies on stem cell therapy show the efficacy of POI treatment, the underlying molecular mechanism and safety are not fully understood. The possible therapeutic mechanisms are involved in migration, antiapoptotic effects, antifibrotic activity, anti-inflammation, immunoregulation, and antioxidative stress [7]. An accumulating body of data indicates that melatonin has been discovered to mediate reproduction through interactions with melatonin receptor type 1 (MT1) and type 2 (MT2) in ovaries [20–22]. Melatonin combines with MT1 and MT2 to reduce levels of reactive oxygen species (ROS) [23, 24], increase glutathione S-transferase (GST) and glutathione peroxidase (GPx) activities, and inhibit glutathione (GSH) [25] and plasma Se levels [26]. A previous study showed that melatonin inhibited apoptosis and increased MT2, superoxide dismutase (SOD), and GPx4 expression, and an antagonist of MT1 and MT2 blocked these protective effects in follicular atresia and porcine GCs [27]. Another study reported that knockdown of MT1 in mouse GCs upregulated apoptosis and impeded proliferation. However, continuous follicle-stimulating hormone (FSH) administration inhibited apoptosis and improved proliferation observably [28]. Moreover, a recent study detected that melatonin had antioxidant effects that mediate cytoprotective activity against cisplatin-induced ovary damage by the MT1 receptor [29]. Although the reproductive protection role of melatonin has been widely explored in various animal models, these studies have focused on melatonin and its relevant receptors. Therefore, it is still necessary to evaluate whether other materials such as fMSCs can regulate the MT1 receptor, which could maintain follicle number and sex hormone levels by changing the level of ROS, related antioxidant enzyme activity, and the balance between apoptotic and antiapoptotic activities in the ovary.

Therefore, the objectives of this study were to evaluate whether fMSCs could restore ovarian function in POI mice, whether MT1 is involved in protective regulation in POI mice and POI hGCs, and whether the potential ovary protective effects of fMSCs are targeted to MT1.

## Methods

### Isolation and culture of human fetal mesenchymal stem cells (fMSCs)

Fetal liver tissue collection for research purposes was approved by the Research Ethics Board of Suzhou Hospital Affiliated with Nanjing Medical University. Tissues were obtained from legally aborted healthy first trimester fetuses (*n* = 10, range from 10 to 12 weeks). All the donors with written consent for the use of fetal tissue screened negative for hepatitis virus, syphilis, and human immunodeficiency virus.

Human first trimester fMSCs from the livers were isolated and cultured as described previously with some modification [30]. In brief, the fresh fetal liver was collected, minced, and filtered through a nylon filter with a size of 70 μm (BD, USA). The single cells were resuspended in culture medium that included DMEM (Thermo, USA), 10% fetal bovine serum (Thermo, USA), 2 mM l-glutamine, 50 U/ml penicillin, and 50 mg/ml streptomycin (Thermo, USA). Cells were plated into 6-well plates at a density of 10^5^ cells/cm^2^ and incubated in a regular incubator. After 72 h, floating cells were cleared, and the medium was changed every other day. When the cells grew to 80–90% confluence, they were digested with 0.25% trypsin and 1 mM EDTA (Thermo, USA) at 37 °C for 5 min for the experiments. The fMSCs were confirmed to express the MSC markers (CD105, CD29, CD73, and CD90) using flow cytometry. The pluripotency stem cell markers Oct-4, Nanog, and Rex-1 were tested by western blot, and the differentiation ability of adipogenesis, chondrogenesis, and osteogenesis was determined. Third- to fourth-passage fMSCs were used for the experiments.

### Isolation and culture of primary human granulose cells (hGCs)

Primary hGCs were isolated from POI patients and young tubal factor infertility patients (age < 40 years old) undergoing oocyte retrieval for in vitro fertilization as described in our study [12]. Briefly, follicular fluid was collected for research purposes and contained discarded red blood cells and granulosa cells. These cells were removed by centrifugation, and the GCs were purified using density centrifugation with Percoll solution (Sigma, USA). Cell culture medium included DMEM/F12 media (Thermo, USA), 10% fetal bovine serum (Thermo, USA), 50 mg/ml streptomycin, 50 U/ml penicillin (Thermo, USA), 1× GlutaMAX (Thermo, USA), and 100 mg/ml streptomycin sulfate (Thermo, USA).

### Experimental animals

Female ICR mice (*n* = 150), aged from 7 to 8 weeks, were obtained from Nanjing Medical University. Three groups were divided randomly. The first group was the normal group (NG) (*n* = 50) and received no treatment. The second group, the POI group (*n* = 50), received a single intraperitoneal (i.p.) injection of cyclophosphamide (CTX, Sigma-Aldrich, 120 mg/kg injected everyday continued for 2 weeks). The third group, the fMSC group (*n* = 50), received a tail vein injection of approximately 1 × 10^6^ fMSCs 2 weeks after CTX injection. The feeding environment and daily inspection of the mice were the same as in our previous study [12]. The blood was collected at 0, 1, 2, 3, and 4 weeks after the treatment with fMSCs synchronously in the three groups. 0.5 ml blood samples were obtained by retroorbital puncture under anesthesia. After being centrifuged at 4000 r/min for 10 min, blood samples were incubated at room temperature for 1 h. The blood and ovaries were used for ELISA, histological, FACS, qPCR, and western blot analyses.

### Adipogenic, osteogenic, and chondrogenic differentiation

As previously described [10], fMSCs were incubated in human mesenchymal stem cell differentiation kits (adipogenesis, osteogenesis, and chondrogenesis differentiation kits, Thermo Fisher, USA). According to the instructions, adipogenesis was detected by Oil red O solution after 14 days of culture, osteogenesis was tested by the alizarin red dye method after 28 days of culture, and an Alcian blue dye assay was used to test chondrogenesis after 21 days of culture.

### Histological analysis

The three groups of mice (*n* = 10 per group) were euthanized synchronously at 8 weeks after treatment with fMSCs. Ovaries were harvested and fixed in 10% formalin. After 24 h of fixation, increasing concentrations of ethanol dehydrated the ovarian tissue, xylene clarified the ovarian tissue, and the tissues were paraffin-embedded. The paraffin was sectioned (5 μm thick) for H&E staining to observe the morphological structure of the mouse ovaries. Four hierarchical follicles were classified using the accepted morphological criteria [31]. In each ovary, three representative sections were used to count the number of antral follicles and total follicles. Only follicles containing an oocyte were counted.

### ELISA analysis

Plasma from the three groups of mice was collected at 0, 1, 2, 3, 4, and 8 weeks after fMSC treatment. An ELISA kit (Mybiosource, USA) was employed to detect the levels of E2, AMH, and FSH according to the directions. In addition, mouse plasma and hGCs (NG, POI, and fMSCs) with or without fMSC treatment were also harvested to evaluate the levels of malondialdehyde (MDA), lactate dehydrogenase (LDH), SOD, glutathione reductase (GR), catalase (CAT), and GPx using an ELISA kit (Cayman Chemical, USA). In brief, the test plate containing 50 μl serum or supernatant sample per well was wrapped and incubated at 37 °C for 30 min. Then, a wash buffer was used to wash the dried wells five times (10 s per wash). Thereafter, 50 μl HRP-conjugate reagent was added to each well and incubated at 37 °C for 60 min. Wash buffer was used to wash the wells five times (10 s, per wash). Then, 50 μl of substrate A solution and substrate B solution were mixed together at 37 °C for 15 min. Then, 50 μl of stop solution was added to each well. Finally, a spectrophotometer (BioTek, USA) was used to test the light absorbance.

### MT1 gene silencing with RNA interference or the antagonist luzindole

To silence the MT1 gene, MT1 siRNA (MyBioSource, USA) was transfected into normal hGCs as previously described [12, 28]. There were three groups in this study. The first group was normal hGCs without any treatment. The second group, the normal hGCs, was transfected with MT1 siRNA (hGCs-MT1^KD^). The third group was hGCs-MT1^KD^ cells treated with fMSCs. Briefly, fMSCs were seeded on the upper coculture transwell inserts, while hGCs-MT1^KD^ cells were on the bottom of a six-well culture plate (Corning, USA). In brief, the hGCs were seeded in three 6-well plates 72 h prior to transfection, and the confluency reached 50–60%. MT1 siRNA was transfected into hGCs using Dharmafect 1 (GE Healthcare, USA). Fresh culture medium was added after transfection for 6 h, and the hGCs were used for subsequent experiments after 72 h. The efficiency of MT1 gene silencing was verified by western blot analysis.

In addition, the pharmacological modulation of MT1 was analyzed by the administration of the MT1 antagonist luzindole. Briefly, normal hGCs were treated with luzindole at different final concentrations (10 μM, 20 μM, 40 μM, and 80 μM) for 2 weeks. The inhibitory effect of MT1 was verified by western blotting. Administration of 40 μM and 80 μM luzindole efficiently blocked the protein expression of MT1. Therefore, three groups were divided randomly. The first group, the NG group, included normal hGCs without treatment. The second group included normal hGCs treated with luzindole at a final concentration of 40 μM. The third group included normal hGCs treated with luzindole and fMSCs together. fMSCs were seeded on the upper coculture inserts, while hGCs were seeded on the bottom of a six-well culture plate using a transwell system (Corning, USA).

### Flow cytometry analysis

fMSCs were dissociated with 0.05% trypsin-EDTA at 37 °C for 5 min. The ovary tissues were dissected, washed in PBS, and then enzymatically digested to single-cell suspensions (0.25% trypsin-EDTA for 15 min). Single-cell suspensions were passed through a 70-μm nylon filter (BD, USA). After washing with PBS, the collected cells were suspended in buffer (0.1% BSA in PBS). To analyze cell surface markers on fMSCs, the dissociated cells were stained with the following PE-conjugated antibodies: anti-CD105, anti-CD29, anti-CD73, anti-CD90, anti-CD34, and anti-CD45, all purchased from Becton Dickinson and Company (USA). The hGCs and cell suspensions from ovarian tissues were stained with anti-KI67 (BD, USA), anti-ROS (Abcam, USA), or its corresponding isotype control at 4 °C for 30 min. The stained cells were analyzed with a flow cytometer (Beckman, USA) using the manufacturer’s directions.

### RNA extraction and real-time polymerase chain reaction (qPCR)

Total RNA was extracted from the hGCs and ovarian tissues by using the RNeasy mini kit (QIAGEN, USA) and reverse-transcripted to cDNA by using the PrimeScript RT reagent kit (Takara, Japan) as described previously [12]. qPCR on amplified cDNA was performed with Power SYBR Premix Ex Taq (Takara, Japan) using a Thermal Cycler Dice Real Time System (Takara, Japan). The data were analyzed by the 2-ΔΔCt calculation method with glyceraldehyde 3-phosphate dehydrogenase (GAPDH) as the internal reference. The primer sequences used are listed in Additional file 1: Table S1.

### Western blot analysis

fMSCs, hGCs, and ovarian tissues were harvested for protein extraction. Western blotting was performed as previously described [12]. The primary antibodies used for fMSCs were anti-Oct4, anti-Nanog, anti-Rex1, and anti-β-Actin, all purchased from Abcam (USA). The primary antibodies used for hGCs and the ovarian tissues were anti-SURVIVIN, anti-BCL2, anti-CASPASE-3, anti-CASPASE-9, anti-MT1, anti-JNK1, anti-PCNA, anti-AMPK, anti-β-Actin, and anti-GAPDH, all purchased from Abcam (USA).

### Statistical analysis

All results are expressed as the means ± SD. SPSS 19.0 software was used to carry out one-way ANOVA, with *p* < 0.05 considered statistically significantly different.

## Results

### Characterization of fMSCs

**Fig. 1:**
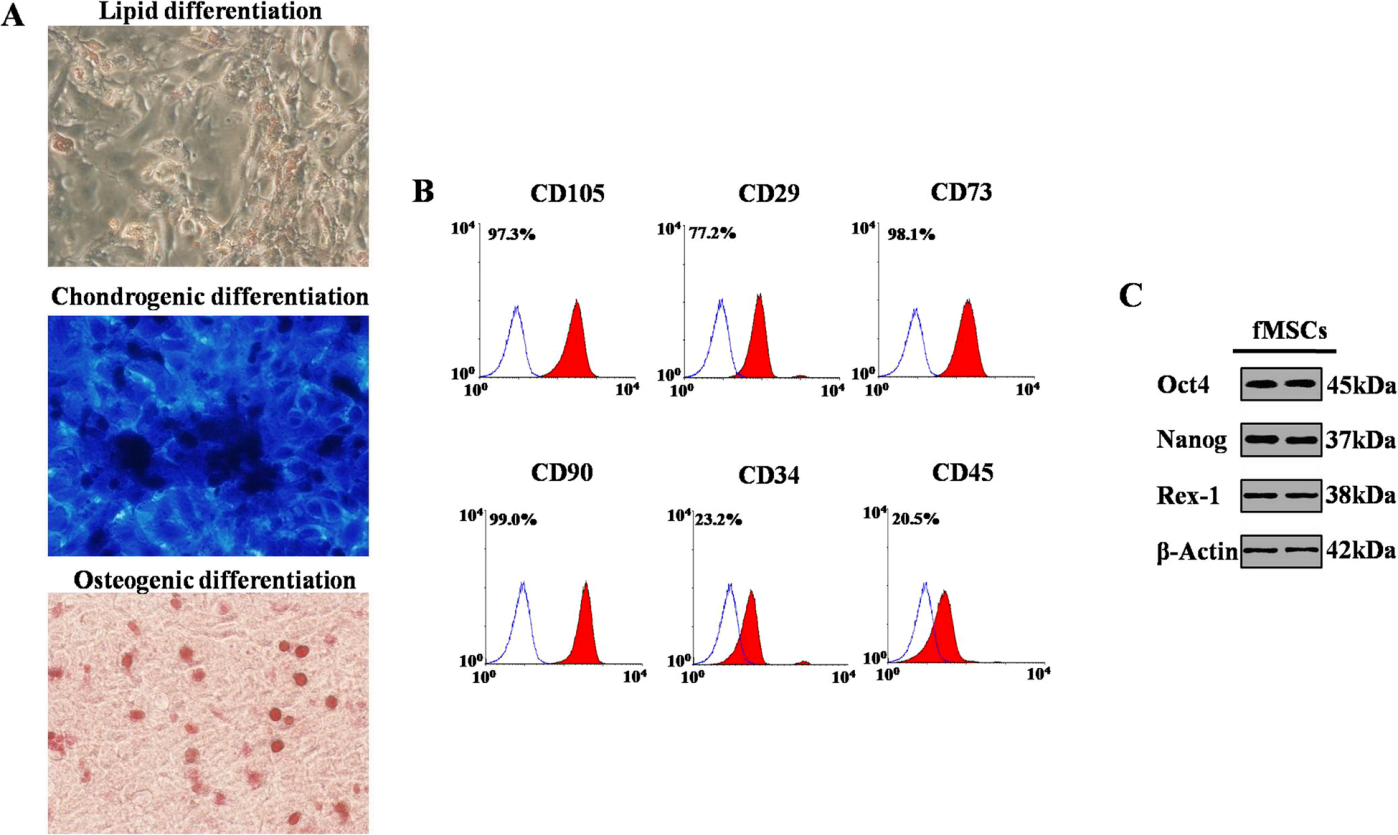
Characteristics of fMSCs. **a** Phenotypes of fMSC differentiation into adipocytes, chondrocytes, and osteocytes by light microscopy. **b** Immunophenotypes of fMSCs by flow cytometry. **c** Western blot analyses of Oct4, Nanog, and Rex-1 in fMSCs. fMSCs fetal mesenchymal stem cells

To confirm whether the cells from first trimester fetal livers have properties of MSCs, western blot, cell differentiation, and FACS analysis were performed. The differentiation potency of these cells was confirmed by adipogenesis, osteogenesis, and chondrogenesis differentiation (Fig. 1a). Then, the various immunophenotypes of the cells were detected by FACS. As shown in Fig. 1b, adherent fetal liver cells were highly expressed in the MSC markers CD105 (97.3%), CD29 (77.2%), CD73 (98.1%), and CD90 (99.0%) and expressed low levels of the hematopoietic markers CD34 (23.2%) and CD45 (20.5%). In addition, western blot assay indicated that the cells expressed the transcription factor markers Oct4, Nanog, and Rex-1 (Fig. 1c).

### Effects of fMSCs on follicle numbers and sex hormone levels in a CTX-induced POI mouse model

**Fig. 2:**
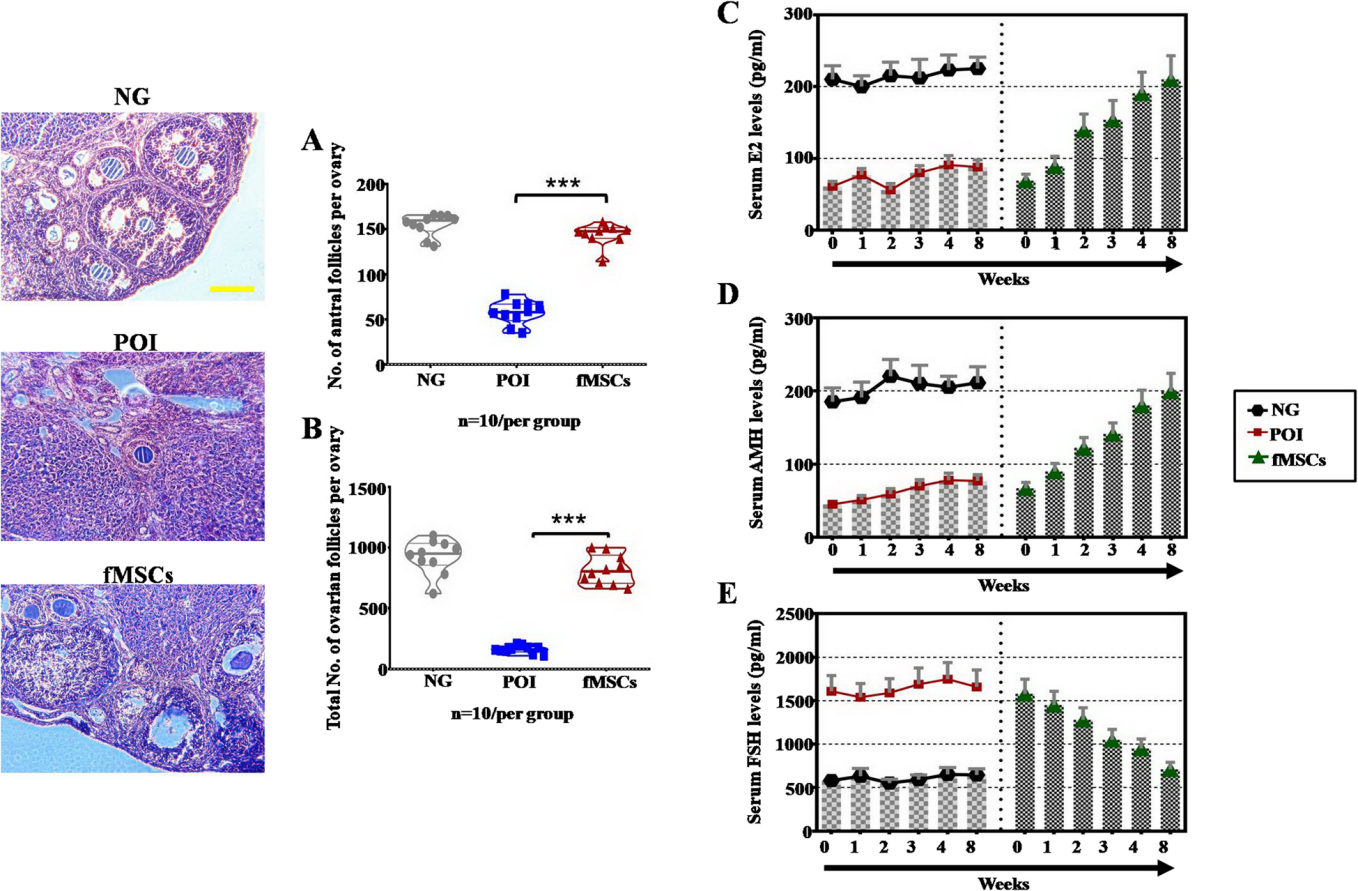
Effects of fMSCs on follicle numbers and sex hormone levels in the POI mouse model. **a** Number of antral follicles at 8 weeks was calculated in NG, POI, and fMSC treatment groups. **b** Number of total follicles at 8 weeks was calculated in NG, POI, and fMSC treatment groups. **c** ELISA analysis of E2 levels was assessed at 0, 1, 2, 3, 4, and 8 weeks in NG, POI, and fMSC treatment groups. **d** ELISA analysis of AMH levels was assessed at 0, 1, 2, 3, 4, and 8 weeks in NG, POI, and fMSC treatment groups. **e** ELISA analysis of FSH levels at 0, 1, 2, 3, 4, and 8 weeks was assessed in NG, POI, and fMSC treatment groups. Data are represented as the mean ± SD. ***p < 0.001 (compared with the POI group)

To better understand the potential therapeutic effects of fMSCs, we compared the number of follicles and the serum levels of sex hormones in mice in different treatment groups. The number of antral follicles in the fMSC group was significantly increased in comparison to that in the POI group after treatment 8 weeks (Fig. 2a). The total follicle numbers exhibited similar results (Fig. 2a). Then, ELISA was used to analyze the levels of E2, AMH, and FSH in serum from the three groups at 0, 1, 2, 3, 4, and 8 weeks after fMSC injection. Compared to levels in the POI group, the levels of E2 and AMH in the fMSC group were elevated similar to those in the NG group in a time-dependent manner (Fig. 2c, d). The level of FSH showed a trend toward a time-dependent decrease in stimuli compared to normal levels (Fig. 2e).

### fMSCs decreased oxidative damage, increased oxidative protection, improved antiapoptotic effects, and inhibited apoptosis in POI hGCs

**Fig. 3:**
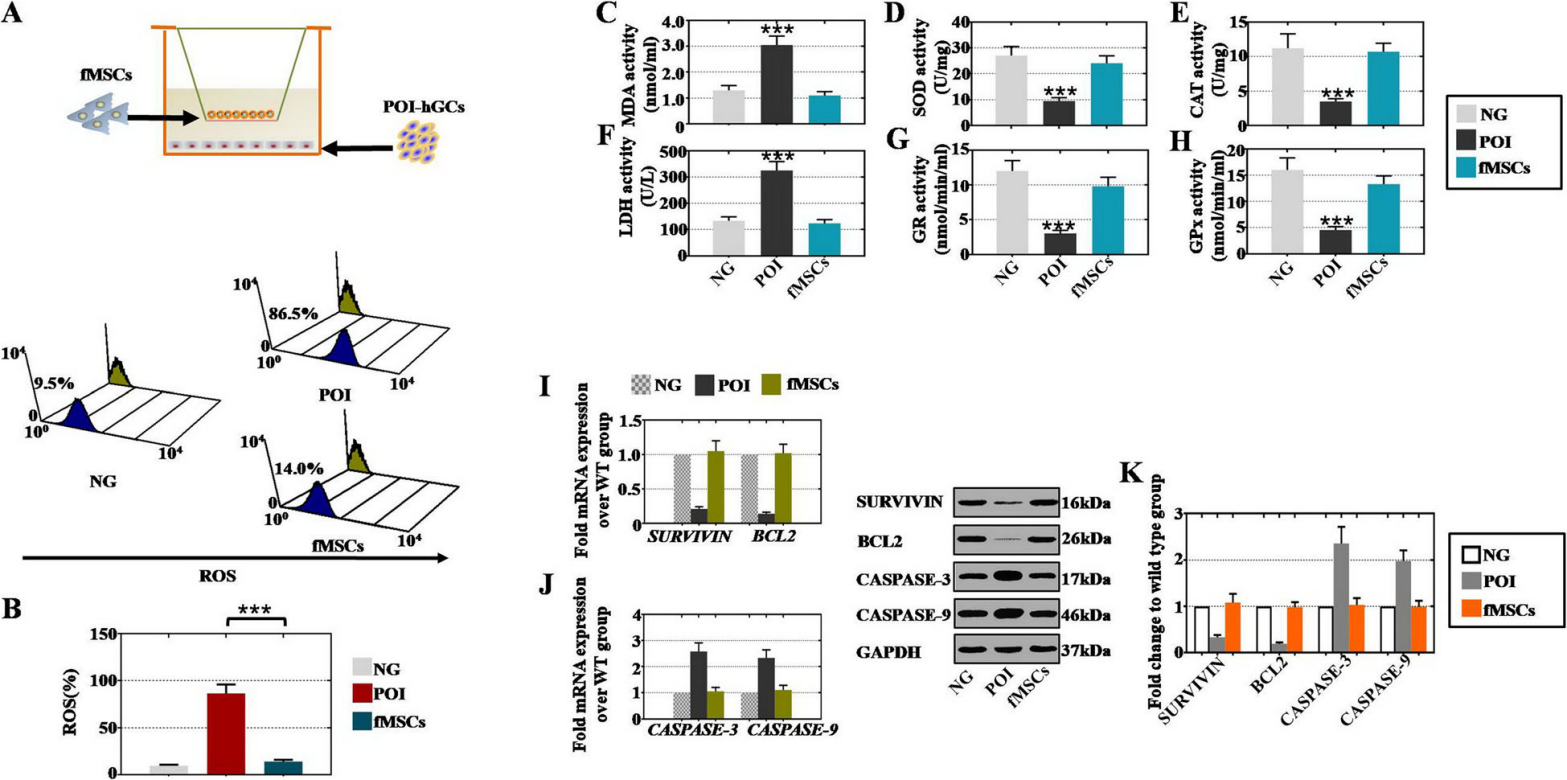
fMSCs decreased oxidative damage, increased oxidative protection, improved anti-apoptosis rates, and inhibited apoptosis in POI hGCs. **a** fMSCs were seeded on the upper coculture inserts, while POI hGCs were seeded on the bottom of a six-well culture plate. **b** FACS analysis of ROS expression at 8 weeks in different treatment groups. **c–h** ELISA analysis of MDA, SOD, CAT, LDH, GR, and GPx levels at 8 weeks in different treatment groups. **i** qPCR analysis of SURVIVIN and BCL2 in different treatment groups. **j** qPCR analysis of CASPASE3 and CASPASE9 in different treatment groups. **k** Western blot analysis of SURVIVIN, BCL2, CASPASE3, and CASPASE9 in different treatment groups. Data are represented as the mean ± SD. ***p < 0.001 (compared with the NG group)

Given that oxidative stress is an inducer of pathological ovarian aging [32], we speculated that the fMSC-mediated improvement in ovarian activity might be attributed to the decreased ROS level. To test this hypothesis, POI hGCs were treated with fMSCs to measure the ROS rate by FACS (Fig. 3a). The ROS rate in the fMSC group (14.0%) was remarkably suppressed compared with that in the POI group (86.5%) as shown in Fig. 3b. We also measured oxidative and antioxidative enzymes and metabolite activity to further confirm the influence of ROS. ELISA was used to quantitatively evaluate MDA, LDH, SOD, GR, CAT, and GPx activities. The activities of SOD, GR, CAT, and GPx in the POI group were decreased significantly, whereas the activities of MDA and LDH were elevated markerly which compared to the NG group. All these enzymes and metabolites were restored nearly to the level of the NG group after fMSC treatment with POI hGCs (Fig. 3c–h).

Thereafter, qPCR was employed to detect the mRNA expression levels of antiapoptotic and apoptotic markers in hGCs in the three groups (NG, POI, and fMSC treatment). At the mRNA level, a more than fourfold decrease in antiapoptotic markers (SURVIVIN and BCL2) was observed in the POI group in comparison to that in the NG group (Fig. 3i), while a more than threefold increase in apoptotic markers (CASPASE-3 and CASPASE-9) was observed in the POI group in comparison to that in the NG group (Fig. 3j). However, these same markers in the fMSC group were rescued nearly to the level of the NG group (Fig. 3i–j). Moreover, a similar trend in protein expression was exhibited in the three groups. fMSCs upregulated antiapoptotic genes and downregulated apoptotic genes at the protein level (Fig. 3k).

### fMSCs decreased oxidative damage, increased oxidative protection, improved anti-apoptosis levels, and inhibited apoptosis levels in a CTX-induced POI mouse model

**Fig. 4:**
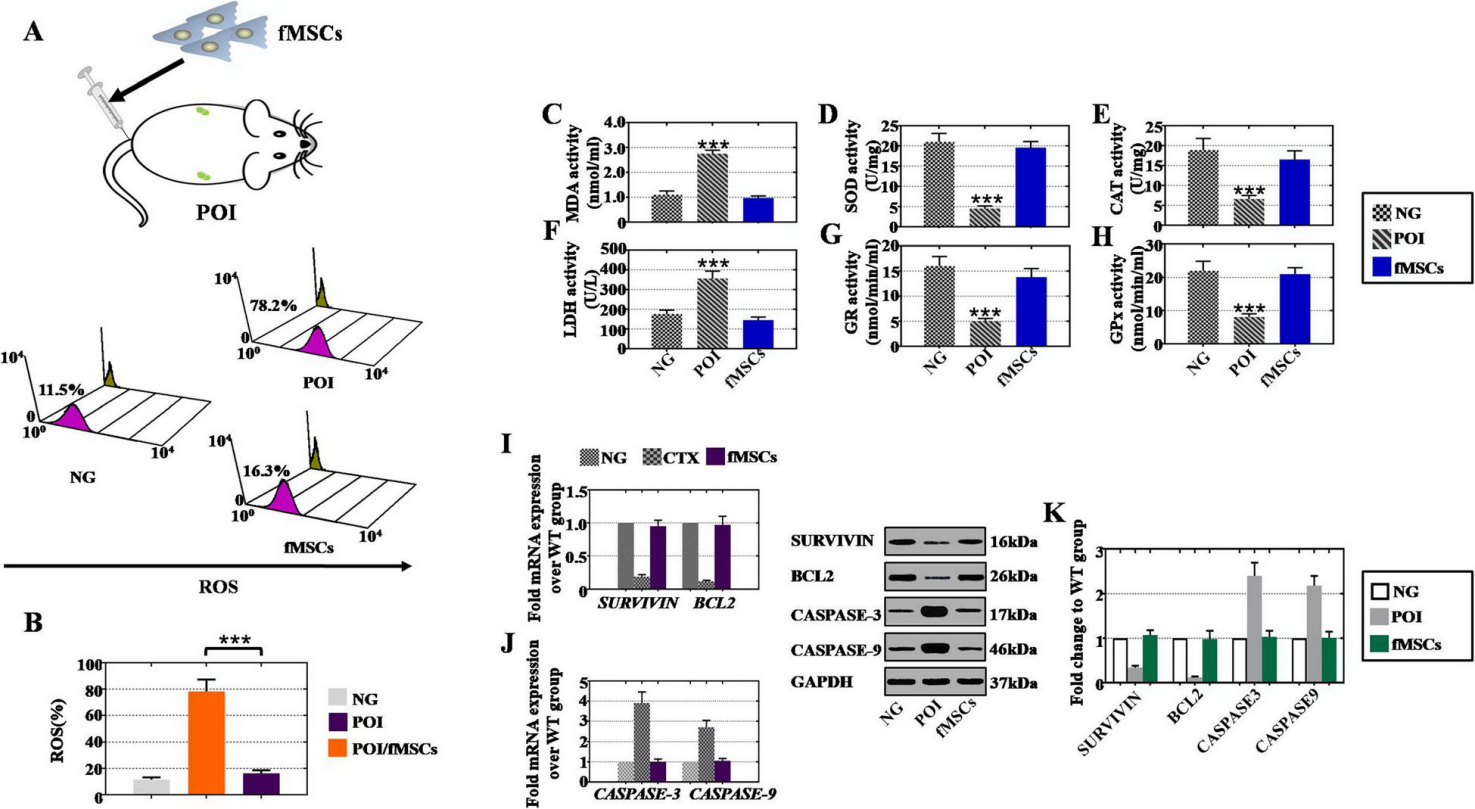
fMSCs decreased oxidative damage, increased oxidative protection, improved anti-apoptosis rates, and inhibited apoptosis in the POI mouse model. **a** fMSCs were injected into the POI mouse model by tail intravenous. **b** FACS analysis of ROS expression at 2 weeks in different treatment groups. **c–h** ELISA analysis of MDA, SOD, CAT, LDH, GR, and GPx levels at 8 weeks in different treatment groups. **i** qPCR detection of SURVIVIN and BCL2 levels in different treatment groups. **j** qPCR analysis of CASPASE-3 and CASPASE-9 levels in different treatment groups. **k** Western blot analysis of SURVIVIN, BCL2, CASPASE-3, and CASPASE-9 in different treatment groups. Data are represented as the mean ± SD. ***p < 0.001 (compared with the NG group)

Because of the in vitro effects of fMSCs on oxidation resistance, anti-apoptosis promotion, and apoptosis inhibition, fMSCs were transplanted into a CTX-induced POI mouse model (Fig. 4a). In line with our results, fMSCs significantly decreased the ROS rate (16.3%) in POI mouse ovaries compared to that of the POI group (78.2%) as shown in Fig. 4b. In addition, the activities of oxidoreductases (MDA and LDH) and antioxidases (SOD, GR, CAT, and GPx) were assessed in the POI group. After fMSC transplantation into POI mouse ovaries, the levels of MDA and LDH were downregulated, and the levels of SOD, GR, CAT, and GPx were upregulated rapidly (Fig. 4c–h). qPCR and western blot analyses also confirmed that the fMSCs improved the antiapoptotic genes (SURVIVIN and BCL2) and repressed the apoptotic markers (CASPASE-3 and CASPASE-9) at the mRNA (Fig. 4i, j) and protein levels in ovaries of POI mice (Fig. 4k).

### fMSCs upregulated MT1, JNK1, PCNA, and AMPK in POI hGCs and the CTX-induced POI mouse model

**Fig. 5:**
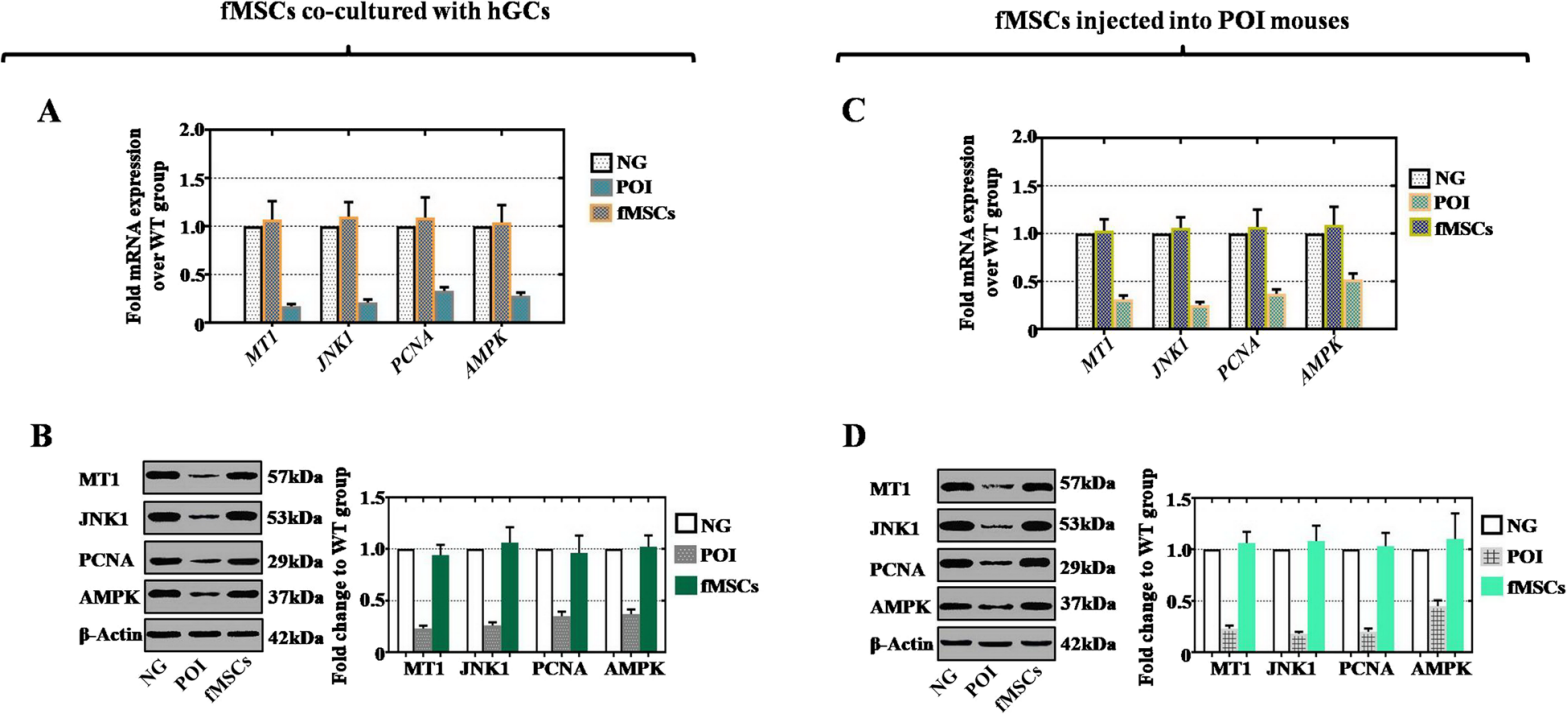
fMSCs upregulated MT1, JNK1, PCNA, and AMPK in vitro and in vivo. **a** qPCR analysis of MT1, JNK1, PCNA, and AMPK in different treatment groups in vitro. **b** Western blot analysis of MT1, JNK1, PCNA, and AMPK in different treatment groups in vitro. **c** qPCR detection of MT1, JNK1, PCNA, and AMPK in different treatment groups in vivo. **d** Western blot detection of MT1, JNK1, PCNA, and AMPK in different treatment groups in vivo

To further explore the potential molecular mechanism, we selected MT1, c-Jun N-terminal kinase 1 (JNK1), proliferation cell nuclear antigen (PCNA), and AMP-activated protein kinase (AMPK) as candidate target genes for fMSCs to improve ovarian function. qPCR analysis was performed to determine the expression of the MT1, JNK1, PCNA, and AMPK genes in the three groups (NG, POI, and fMSCs) in vitro. The mRNA expression levels of MT1, JNK1, PCNA, and AMPK in the fMSC treatment groups were significantly upregulated similar to the levels in the NG group (Fig. 5a). The mRNA expression level of these genes was also elevated significantly in the fMSC group in vivo (Fig. 5c).

In addition, western blot analysis was used to detect the protein expression levels of MT1, JNK1, PCNA, and AMPK in vivo and in vitro. In the POI hGC group, the protein expression levels of MT1, JNK1, PCNA, and AMPK were less than half that in the NG group (Fig. 5b). After incubation with POI hGCs and injection into the POI mouse model, western blot analysis showed that fMSCs remarkably upregulated MT1, JNK1, PCNA, and AMPK expression ex vivo (Fig. 5b) and in vivo (Fig. 5d).

### Therapeutic effect of fMSCs was displayed by targeting MT1

**Fig. 6:**
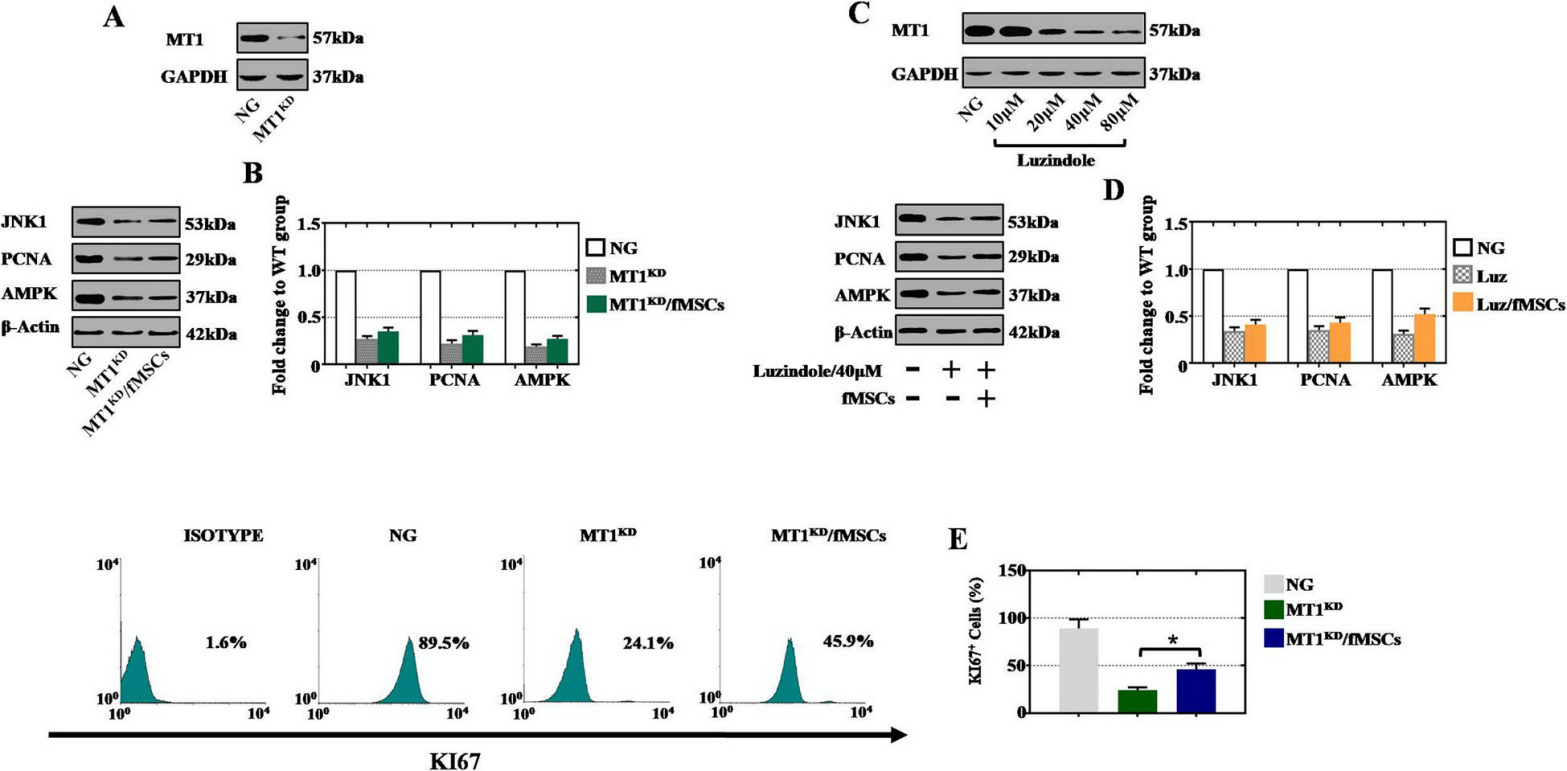
fMSCs restored POI disease by targeting MT1. **a** Western blot analysis of MT1 in hGCs-MT1^KD^ cells. **b** Western blot analysis of JNK1, PCNA, and AMPK expression levels in different treatment groups. **c** Western blot assessment of MT1 after administration of the MT1 antagonist luzindole at different concentrations (10 μM, 20 μM, 40 μM, and 80 μM) in normal hGCs. **d** Western blot assessment of JNK1, PCNA, and AMPK in different treatment groups (luzindole, 40 μM). **e** KI67 expression levels were evaluated in different treatment groups by FACS. Data are represented as the mean ± SD. ***p < 0.05 (compared with the MT1^KD^ group)

Given that melatonin exerts numerous biological functions for ovarian improvement via the MT1 and MT2 receptors of the G-protein-coupled superfamily, the molecular mechanism of MT1 was further evaluated by MT1 siRNA transfection and MT1 antagonist luzindole treatment. The knockdown efficiency of MT1 was detected by using western blotting. After MT1 was knocked down in normal hGCs, the low MT1 level was tested as shown in Fig. 6a. The results indicated that hGCs-MT1^KD^ cell lines were established successfully. To explore the regulatory role of MT1 in the proliferation of hGCs, incubation of fMSCs with the MT1^KD^ hGCs was utilized to detect the efficacy in the present experiment. Western blot analysis revealed that JNK1, PCNA, and AMPK genes at the protein level were downregulated in the MT1^KD^ group, whereas there were no significant changes after hGCs-MT1^KD^ coculture with fMSCs (Fig. 6b). To further investigate the MT1 mechanism, the MT1 antagonist luzindole at different concentrations (10 μM, 20 μM, 40 μM, and 80 μM) was exogenously supplemented into normal hGCs. As Fig. 6c shows, the administration of 40 μM and 80 μM antagonist blocked the protein expression of MT1 efficiently. Therefore, the expression levels of JNK1, PCNA, and AMPK were examined at a concentration of 40 μM, and our results showed that fMSCs did not recover gene expression after antagonist treatment (Fig. 6d).

In addition, we further explored whether fMSCs improved the proliferation rate of hGCs-MT1^KD^. FACS analysis indicated that the percentage of Ki-67^+^ hGCs was sharply decreased to 24.1% in the MT1^KD^ group, whereas the percentage was 89.5% in the NG group (Fig. 6e). The percentage of KI67^+^ hGCs was slightly upregulated to 45.9% in the MT1^KD^/fMSCs group as shown in Fig. 6e.

## Discussion

Due to infertility, POI causes a feeling of sadness and depression in many couples and families. The main pathogenesis of POI disease is elaborated in depth, which is convenient for treatment. However, the etiology of POI remains unclear in the large majority of cases. Although almost all of the studies point to the efficacy of stem cells in POI treatment, the potential mechanisms are not fully understood. The findings from this study demonstrate that fMSC treatment attenuates ovarian damage in a CTX-induced POI mouse model and promotes proliferation of POI hGCs by regulating MT1. To our knowledge, this is the first study to investigate the interaction between fMSCs derived from the liver and POI. The beneficial effects of fMSCs in restoring ovarian function in POI and the potential mechanisms are discussed below.

Although MSCs derived from various cellular sources make them attractive for treating POI disease [6–8], there is no report that discusses the therapeutic effect of MSCs derived from the fetal liver on POI treatment. The current investigation of this effect showed that the CTX-induced POI mouse model treated with fMSCs not only improved the antral follicles and total follicle numbers at four stages but also increased the sex hormone levels to nearly the normal levels (Fig. 2). The disorder of oxidative stress has been implicated in the causal pathways for POF [33, 34]. The generation of ROS plays a role in cellular metabolism under normal physiological conditions, whereas increased ROS levels directly oxidize protein and DNA or indirectly activate intracellular signaling pathways that induce cellular damage [35–37]. In this study, we found that the ROS level was downregulated in the POI mouse model and POI hGCs after treatment with fMSCs (Figs. 3 and 4). LDH, a key regulator of glycolysis, and MDA, a major end product of membrane lipid peroxidation, were increased in vivo and in vitro in the POI model, while they were decreased after fMSC treatment (Figs. 3 and 4). The activities of SOD, GR, CAT, and GPx were inhibited observably in the in vivo and in vitro POI models, but they were elevated after the fMSC treatment (Figs. 3 and 4). These findings suggest that multitargeted antioxidant properties of fMSCs might defend against ovarian damage from oxygen radicals. Although stem cell therapy improves ovarian function in POI by mediating oxidative stress, which has not been reported, BMSCs did have an effective therapeutic function in colitis disease by suppressing oxidative stress [38], and melatonin effectively protected ovarian function against cisplatin-induced ovarian damage by reducing apoptosis, ROS production, and mitochondrial damage [29].

Oxidative stress has been considered an important apoptosis mediator, and the fate of ovarian cells is ultimately determined by the homeostasis of pro- and antiapoptotic genes [39]. Highly precise apoptotic and antiapoptotic regulation are involved in intrinsic and extrinsic pathways [40, 41]. The BCL2 family plays a vital role in the intrinsic pathway [42]. Apoptosis and antiapoptotic proteins assemble and enter the mitochondria, triggering the release of cytochrome c to activate CASPASE-9 and CASPASE-3, which causes DNA damage and cell apoptosis [43]. The current study demonstrated that the apoptotic markers BCL2 and SURVIVIN were upregulated and the antiapoptotic markers CASPASE-3 and CASPASE-9 were downregulated in the POI mouse model and in POI hGCs treated with fMSCs (Figs. 3 and 4). In line with our findings, BMSCs resisted the apoptosis of GCs by increasing the c-myc gene and decreasing the p21 and bax genes to improve ovarian morphology and function in POF rats [44], and umbilical cord MSCs inhibited apoptosis of POI rat ovaries by decreasing CASPASE-3 to restore ovarian function and structure [45]. These findings suggest that fMSCs might improve fertility preservation in POI disease by improving the antiapoptotic level and inhibiting apoptosis.

Many signaling molecules, receptors, and pathways participate in the recovery of ovarian function. Currently, a growing body of evidence shows that melatonin and MT1 improve oocyte and embryo early development and regulate the level of sex hormones [22, 46, 47]. JNK is a part of the mitogen-activated protein kinase (MAPK) signaling pathway mediating cellular responses to various stress stimuli, and its subfamily includes the JNK1, JNK2, and JNK3 genes [48]. JNK modulates a variety of biological functions, including proliferation, apoptosis, migration, differentiation, and inflammation [49–51]. PCNA is the major regulator of replication fidelity and DNA damage repair, and it is essential for normal cellular proliferation [52]. AMPK is a vital intracellular energy sensor that promotes catabolic pathways to generate more ATP, suppresses anabolic signaling in low ATP conditions, and mediates the coordination of cell growth and metabolism [53, 54]. In this study, we observed a significant decrease in the expression of MT1, JNK1, PCNA, and AMPK by qRCR and western blotting in the POI mouse model and POI hGCs, whereas these genes were markedly increased after treatment with fMSCs (Fig. 5). Together, these data highlight that MT1, JNK1, PCNA, and AMPK may participate in cellular proliferation, apoptosis, migration, and ovary damage. Although stem cell therapy restores ovarian function in POI by directly mediating MT1, JNK1, PCNA, and AMPK, which has not been previously reported, a supporting study revealed that melatonin effectively elevated the percentage of PCNA-positive granulosa and theca cells [29] and that the radiation-POF mouse model treated with niacin highly expressed PCNA in the GCs [55]. In a recent study, human ovaries and hGCs were exposed to cisplatin in vitro, which elevated the levels of the phosphorylated forms of SAPK/JNK and triggered apoptosis [56]. A previous study demonstrated that MT1, AMPK, and p-AMPK were upregulated in the ovaries of melatonin-treated mice compared to those of untreated mice, which is similar to the results of our study [57].

Given that melatonin, through interaction with membrane receptors (MT1 and MT2), plays a vital role in reproduction, our aim was to explore whether cellular materials such as fMSCs can regulate the MT1 receptor to preserve the fertility of ovaries by influencing ROS and related antioxidant enzyme activity, proliferation, or apoptosis rate in the ovary niche during oogenesis. When normal hGCs inhibited MT1 expression effectively by RNA interference and chemical inhibitors, the protein levels of JNK1, PCNA, and AMPK were not markedly increased after fMSC treatment, and the percentage of cells with the proliferation marker KI67 was elevated significantly when hGCs-MT1^KD^ were treated with fMSCs (Fig. 6). This phenomenon may indicate that MT1 is not the only regulator of proliferation.

**Fig. 7:**
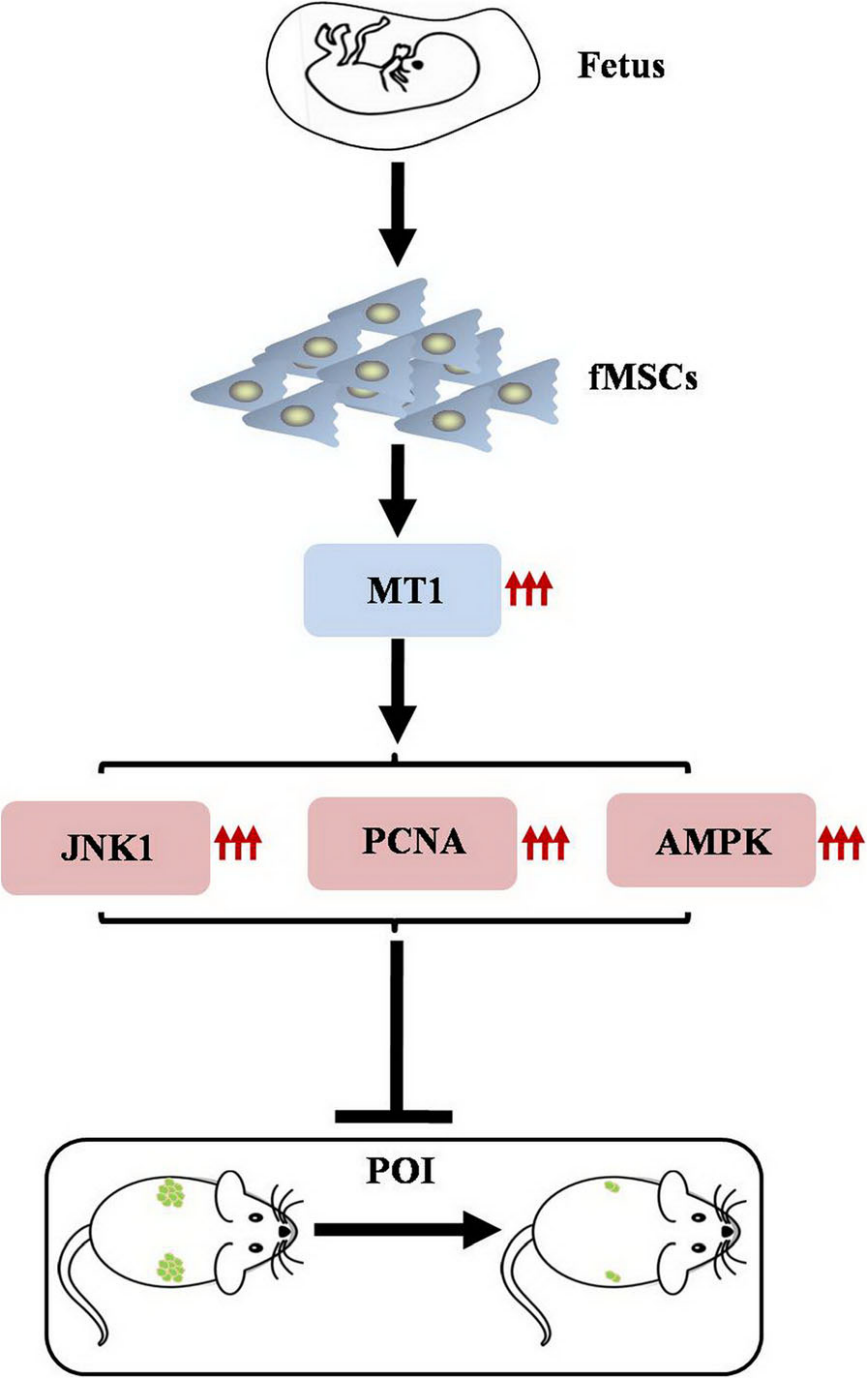
Proposed model for fMSCs improved ovarian function of POI mice through regulating MT1 pathway

Our results are in agreement with an earlier study that showed that silencing of MT1 by RNA interference led to increased apoptosis in mGCs, whereas the apoptotic effect was inhibited after mGCs-MT1^KD^ administration with FSH [28]. Similar to our findings, a previous experiment indicated that administration of the MT1/MT2 receptor antagonist (luzindole) inhibited the therapeutic effect of melatonin on chemotoxic ovary damage [29]. We observed similar results in our study, and fMSCs were unable to increase JNK1, PCNA, and AMPK expression after inhibition of MT1 (Fig. 7).

## Conclusion

In summary, fMSCs can be used to recover ovarian function. Furthermore, we explore the potential mechanisms. First, fMSCs exert antioxidant effects through their downregulation of ROS, MDA, and LDH and upregulation of SOD, GR, CAT, and GPx levels in vitro and in vivo. In addition, fMSCs display proliferation effects due to their elevation of SURVIVIN and BCL2 and repression of CASPASE-3 and CASPASE-9. Moreover, fMSCs might enhance the biological activity of hGCs by mediating MT1 and its downstream genes. These discoveries raise the possibility of using fMSCs to treat POI disease in humans. Furthermore, we elaborated the mechanism by which MT1 is a preponderant therapeutic target for restoring ovarian function.

## Supplementary information

**Additional file 1: Table S1.** Designations, sequences, and the sizes of real-time PCR amplicons.

## Data Availability

All the data generated or analyzed during this study are included in this published article.

## Abbreviations

POI: Premature ovarian insufficiency
POF: Premature ovarian failure
fMSC: Fetal mesenchymal stem cells
MT1: Melatonin receptor 1
NG: Normal group
CTX: Cyclophosphamide
hGC: Human granulosa cell
E2: Estradiol
AMH: Anti-Mullerian hormone
FSH: Follicle-stimulating hormone
ROS: Reactive oxygen species
MDA: Malondialdehyde
SOD: Superoxide dismutase
CAT: Catalase
LDH: Lactate dehydrogenase
GR: Glutathione reductase
GPx: Glutathione peroxidase
JNK1: c-Jun N-terminal kinase 1
PCNA: Proliferation cell nuclear antigen
AMPK: AMP-activated protein kinase
ELISA: Enzyme-linked immunosorbent assay
FACS: Flow cytometry
FACS: Fluorescence-activated cell sorting
HE: Hematoxylin and eosin
qPCR: Quantitative polymerase chain reaction
GAPDH: Glyceraldehyde 3-phosphate dehydrogenase
