# Supplementary material for: Fetal liver mesenchymal stem cells restore ovarian function in premature ovarian insufficiency by targeting MT1
Source: Stem Cell Res Ther. 2019 Nov 29;10:362. doi: 10.1186/s13287-019-1490-8 (PMC6884777; doi:10.1186/s13287-019-1490-8)
Supplement: Supplementary file 1 — Additional file 1: Table S1. Designations, sequences, and the sizes of real-time PCR amplicons. [file 13287_2019_1490_MOESM1_ESM.doc]

**Supplemental Table 1 Designations, sequences, and the sizes of real-time PCR amplicons**

| **Name** | **Sequence from 5'-3'** | **Size (bp)** |
| --- | --- | --- |
| SURVIVIN (H) Fw | AGGGACCATGCAGGGCAAC | 243 |
| SURVIVIN (H) Rev | TAAATGGCCACCACCAGGTC |
| BCL2 (H) Fw | CTTTGAGTTCGGTGGGGTCA | 187 |
| BCL2 (H) Rev | GAAATCAAACAGAGGCCGCA |
| CASPASE3 (H) Fw | ATTTGGAACCAAAGATCATACATGG | 185 |
| CASPASE3 (H) Rev | TTCCCTGAGGTTTGCTGCAT |
| CASPASE9 (H) Fw | GTGGCGCGGAATGGAT | 174 |
| CASPASE9 (H) Rev | GAAACGAAGCCAGCATGTCC |
| MT1 (H) Fw | AAAGGGGCATCGGAGAAGTG | 125 |
| MT1 (H) Rev | GCAAAGGGGTCAAGATTGTAGC |
| JNK1 (H) Fw | CTGAAGCAGAAGCTCCACCA | 168 |
| JNK1 (H) Rev | GCTGCACCTGTGCTAAAGGA |
| PCNA (H) Fw | GCCAGAGCTCTTCCCTTACG | 87 |
| PCNA (H) Rev | TAGCTGGTTTCGGCTTCAGG |
| AMPK (H) Fw | GGGTGAAGATCGGCCACTAC | 73 |
| AMPK (H) Rev | TTGCCAACCTTCACTTTGCC |
| GAPDH (H) Fw | GAAGGTCGGAGTCAACGGATTT | 223 |
| GAPDH (H) Rev | CTGGAAGATGGTGATGGGATTTC |
| SURVIVIN (M) Fw | TGGGAGAGAGCAGGCAAATTA | 188 |
| SURVIVIN (M) Rev | ACACCCCAGCCAATCAAGTC |
| BCL2 (M) Fw | CTTTGAGTTCGGTGGGGTCA | 152 |
| BCL2 (M) Rev | GTTCCACAAAGGCATCCCAG |
| CASPASE3 (M) Fw | GAGCTTGGAACGGTACGCTAA | 118 |
| CASPASE3 (M) Rev | GAGTCCACTGACTTGCTCCC |
| CASPASE9 (M) Fw | GAGCTTCGAGAACTACCGCA | 84 |
| CASPASE9 (M) Rev | GTATTCCCGCGATCCCCTTC |
| MT1 (M) Fw | TCACCACGACTTCAACGTCC | 86 |
| MT1 (M) Rev | CAGTTGGGGTCCATTCCGAG |
| JNK1 (M) Fw | CTTCAGAAGCAGAAGCCCCA | 163 |
| JNK1 (M) Rev | TGTGCTAAAGGAGACGGCTG |
| PCNA (M) Fw | AAAGATGCCGTCGGGTGAAT | 179 |
| PCNA (M) Rev | TGGTTACCGCCTCCTCTTCT |
| AMPK (M) Fw | GCTTTATTGTGCGGCCCAG | 124 |
| AMPK (M) Rev | GCCAGGCAGGTGCAATTAAC |
| GAPDH (M) Fw | TTCCAGTATGACTCTACCCACGGCA | 137 |
| GAPDH (M) Rev | GCACCAGCATCACCCCATTTG |

**H=Human; M=Mouse.**
